# THE ROLE OF A MONOCLONAL GAMMOPATHY OF UNDETERMINED SIGNIFICANCE DIAGNOSIS IN HEALTHCARE UTILIZATION

**DOI:** 10.1093/geroni/igac059.590

**Published:** 2022-12-20

**Authors:** Maira Castaneda-Avila, Kate Lapane, Mara Epstein

**Affiliations:** University of Massachusetss Chan Medical School, Shrewsbury, Massachusetts, United States; University of Massachusetts Chan Medical School, Worcester, Massachusetts, United States; Meyers Health Care Institute, Department of Medicine, UMass Chan Medical School, Worcester, Massachusetts, United States

## Abstract

Monoclonal Gammopathy of Undetermined Significance (MGUS) is an understudied precursor of multiple myeloma (MM), the second most prevalent hematologic malignancy in the US. MGUS is incidentally diagnosed, and its significance is unclear as only 1% per year transition to MM. MGUS is highly prevalent among adults aged ≥ 50 years. In this presentation, we will review mixed-method approaches. Using healthcare claims and electronic health records from patients in central Massachusetts, we applied group-based trajectory modeling and conditional Poisson regression. These analyses were complemented by a qualitative analysis of in-depth interviews with providers and MGUS patients. Together, these methodologies provided a comprehensive evaluation of the impact of MGUS on healthcare utilization in older adults. The qualitative analysis provided a better understanding of the patient and provider factors influencing healthcare utilization after an MGUS diagnosis. The presentation will highlight how the use of these methodologies provide different perspectives among understudied premalignant conditions.

